# Influence of steroidal implants and zinc sulfate supplementation on growth performance, trace mineral status, circulating metabolites, and transcriptional changes in skeletal muscle of feedlot steers

**DOI:** 10.1093/jas/skae154

**Published:** 2024-06-03

**Authors:** Dathan T Smerchek, Emma L Rients, Amy M McLaughlin, Kara J Thornton, Stephanie L Hansen

**Affiliations:** Department of Animal Science, Iowa State University, Ames, IA 50011, USA; Department of Animal Science, Iowa State University, Ames, IA 50011, USA; Department of Animal Science, Iowa State University, Ames, IA 50011, USA; Department of Animal, Dairy and Veterinary Science, Utah State University, Logan, UT 84322, USA; Department of Animal Science, Iowa State University, Ames, IA 50011, USA

**Keywords:** cattle, feedlot, implant, zinc

## Abstract

Angus-cross steers (*n* = 144; 362 kg ± 20.4) were used to determine the effect of Zn and steroidal implants on performance, trace mineral status, circulating metabolites, and transcriptional changes occurring in skeletal muscle. Steers (*n* = 6 per pen) were stratified by body weight (**BW**) in a 3 × 2 factorial. GrowSafe bunks recorded individual feed intake (steer as experimental unit; *n* = 24 per treatment). Dietary treatments (**ZINC**; eight pens per treatment) included supplemental Zn as ZnSO_4_ at 1) 0 (analyzed 54 mg Zn/kg DM; **Zn0**); 2) 30 mg/kg DM (**Zn30**); 3) 100 mg Zn/kg DM (**Zn100**). After 60 d of Zn treatment, steers received a steroidal implant treatment (**IMP**) on day 0: 1) no implant; **NO**; or 2) high-potency combination implant (TE-200, Elanco, Greenfield, IN; 200 mg TBA, 20 mg E_2_; **TE200**). BWs were taken at days −60, 0, and in 28 d increments thereafter. Liver biopsies for TM analysis and blood for TM, serum glucose, insulin, nonesterified fatty acids (**NEFA**), urea-N, and IGF-1 analysis were collected on days 0, 20, 40, and 84. Glucose, NEFA, and insulin were used to calculate the revised quantitative insulin sensitivity check index (RQUICKI). Linear and quadratic effects of ZINC were evaluated in SAS 9.4. Means for IMP were separated using the LSMEANS statement with the PDIFF option. Day −60 BW was a covariate for performance and carcass data. Growth performance, plasma, liver, and metabolite data were analyzed as repeated measures. TE200 tended to decrease plasma Zn by 8.4% from days 0 to 20 while NO decreased by 3.6% (IMP × day*; P *= 0.08). A tendency for a ZINC × day effect on G:F was noted (*P* = 0.06) driven by Zn30 and Zn100 decreasing significantly from period 0-28 to period 28-56 while Zn0 was similar in both periods. An IMP × day effect was noted for RQUICKI where (*P* = 0.02) TE200 was greater on day 40 compared to NO cattle, but by day 84 RQUICKI was not different between TE200 and NO. On day 20, increasing Zn supplementation linearly increased mRNA abundance (*P* ≤ 0.09) of protein kinase B (AKT1), mammalian target of rapamycin (**mTOR**), matrix metalloproteinase 2 (**MMP2**), and myogenic factor 5 (**MYF5**). In this study, Zn and implants differentially affected genes related to energy metabolism, satellite cell function, and TM homeostasis on days 20 and 84 postimplant. These results suggest steroidal implants increase demand for Zn immediately following implant administration to support growth and may influence insulin sensitivity in finishing cattle.

## Teaser

Increasing Zn supplementation differentially affects genes related to muscle growth, energy metabolism, satellite cell function, and TM homeostasis. Steroidal implant administration decreases plasma Zn concentration, indicating increased Zn demand during periods of rapid growth.

## Introduction

Total cattle inventory has declined over the past 40 yr; however, there has been a general increase in total pounds of beef produced (Drouillard, 2018). High-potency combination steroidal implants containing estradiol (E_2_) and trenbolone acetate (**TBA**) consistently improve growth rate, feed efficiency, and carcass leanness (Preston, 1999), ultimately contributing to the economic and environmental sustainability of the beef industry (Capper and Hayes, 2012). Nutritional approaches to further optimize this technology represent a valuable contribution to the beef industry.

It is well understood that steroidal implants initiate increased skeletal muscle hypertrophy in part via increased protein synthesis (Johnson and Beckett, 2014). Zinc, the most utilized TM in biological processes, may support steroidal implant-induced growth through synergism with energy metabolism and protein synthesis pathways (Nimmanon et al., 2017). Supplementation up to 150 mg Zn/kg DM from ZnSO_4_ results in improved growth performance in the first 18 d after steers are administered a high-potency implant (Messersmith and Hansen, 2021a). We have also noted consistent decreases in plasma Zn concentration following implantation with a high-potency combination steroidal implant (Messersmith, 2018; Messersmith et al., 2022; Reichhardt et al., 2022).

However, the mechanisms by which Zn positively influences steroidal implant-induced growth are poorly understood. Thus, the objective of this study was to determine how increasing supplemental Zn concentration affects steroidal implant-induced growth, carcass characteristics, TM status, relevant circulating metabolites, and mRNA abundance of genes related to skeletal muscle growth and TM metabolism. The hypothesis was that increasing supplementation of Zn and steroidal implant administration would improve growth performance and carcass characteristics in implanted cattle and would influence circulating metabolites related to energy metabolism as well as genes related to skeletal muscle growth.

## Materials and Methods

All procedures and protocols were approved by the Iowa State University Institutional Animal Care and Use Committee (IACUC-20-127).

### Experimental design

Angus-cross steers (*n* = 144; 362 kg ± 20.4) were utilized in a study conducted at the Beef Nutrition Farm (BNF) located in Ames, IA from early November 2021 to late April 2022. To accommodate sampling logistics, steers were assigned to two blocks (*n* = 72 steers per block; *n* = 12 pens per block). Blocks started on trial with a 14-d stagger where identical sampling and experimental procedures occurred within blocks at identical intervals during the study. Steers were assigned to block by weight to account for the 14-d stagger to achieve similar body weight (**BW**) at equal days postimplant. Steers were stratified by BW and randomly assigned to pens within block. Pens were equipped with GrowSafe feed bunks (*n* = 1 GrowSafe feed bunk per pen of six steers; GrowSafe Systems Ltd., Airdire, AB, Canada). Individual radio frequency tags in the ear of an individual steer allowed for individual steer recognition by the GrowSafe bunk. GrowSafe bunks record feed disappearance and associate it with the corresponding individual radio frequency tags and relay data from the bunk to GrowSafe software. Thus, individual intake data were recorded for each animal in each pen. Steers were stratified by BW into a 3 × 2 factorial design. Dietary treatments (**ZINC**; eight pens per treatment; Zn as ZnSO_4_) included: 1) no supplemental Zn (analyzed 54 mg Zn/kg DM; **Zn0**), 2) 30 mg supplemental Zn/kg DM (**Zn30**), 3) 100 mg supplemental Zn/kg DM (**Zn100**). Dietary treatments began 60 d prior to implant and continued until cattle were harvested. Within ZINC treatment, steers received a steroidal implant treatment on day 0: 1) no implant, **NO**; or 2) high-potency combination implant (Component TE-200 with Tylan, Elanco, Greenfield, IN; 200 mg TBA, 20 mg E2; **TE200**). All steers within a given pen received the same implant treatment. Individual steer BW were recorded at study initiation (days −61 and −60) prior to placement in the final study pen as well as days −1, 0 (day of implant), 28, 56, 89, and 90. On day 91 steers were harvested at a commercial abattoir (National Beef, Tama, IA) via industry-accepted practices. Cattle were harvested at a commercial abattoir (National Beef, Tama, IA) via industry-accepted practices. Trained National Beef personnel collected HCW on the day of harvest. Ribeye area (**REA**), 12th rib fat (**RF**), and marbling data were collected following a 48-h chill. Yield grade data represent the abattoir assigned grade and were not calculated via the USDA yield grade calculation due to lack of kidney, pelvic, and heart fat data. Equations from Guiroy et al. (2002) were used to estimate empty body fat percentage (**EBF**) using carcass measurements including 12th rib fat thickness (**FT**), hot carcass weight (**HCW**), USDA quality grade, and longissimus muscle area.

### Dietary management

Cattle were fed a roughage-based diet from days −60 to −38, transitioned to a high-concentrate diet, and on finishing diet for 23 d before implant. Cattle were fed treatment diets (Table 1) once daily at approximately 0800 hours. Steers were fed ad libitum during the entirety of the study with bunks managed to ensure feed remained in GrowSafe bunks at the time of daily feed calls. Dietary treatments were included in the total mixed ration (**TMR**) as a premix with dried distillers grains plus solubles as a carrier. Water was provided ad libitum throughout the study via automatic waterers available in each pen. Water tanks were checked daily and cleaned by feedlot personnel as needed to ensure a constant and clean water supply to the cattle.

**Table 1.** Finishing diet composition^1^

	% DM basis		
	Zn0	Zn30	Zn100
Ingredient			
Dry-rolled corn	45	45	45
Sweet Bran	20	20	20
Corn silage	15	15	15
DDGS	15	10	10
Zn30 Premix^2^	—	5	—
Zn100 Premix^2^	—	—	5
Basal premix^2^^,^^3^	5	5	5
Analyzed composition, % DM			
Crude protein^4^	15.5	15.5	15.5
NDF^4^	21.79	21.79	21.79
Ether extract^4^	4.86	4.86	4.86
NEm, Mcal/kg^5^	2.07	2.07	2.07
NEg, Mcal/kg^5^	1.41	1.41	1.41
Zn, mg/kg^6^	54	83	157

^1^Dietary treatments (ZINC; eight pens per treatment) included supplemental Zn as ZnSO_4_: no supplemental Zn (**Zn0**); 2) 30 mg supplemental Zn/kg DM (**Zn30**); 3) 100 mg supplemental Zn/kg DM (**Zn100**). Average analyzed Cu, Fe, and Mn concentrations contained in treatment diets were 16, 101, and 36 mg/kg DM, respectively.

^2^Treatment premix and basal utilized DDGS as a carrier. Basal provided as % DM; limestone (1.5%), Rumensin (0.0135%), and salt (0.31%). Trace minerals and vitamins provided per kg of DM: 0.15 mg Co (cobalt carbonate), 20 mg Cu (copper sulfate), 0.1 mg Se (sodium selenite), 0.5 mg I (calcium iodate), 20 mg Mn, (Mn sulfate), and Vitamin A 2,200 IU.

^3^Provided vitamins at 2016 NASEM recommendations.

^4^Nutrient composition values analyzed by Dairyland Laboratories (Arcadia, WI).

^5^Calculated from tabular energy values.

^6^Analyzed values measured by inductively coupled plasma optical emission spectrometry (ICP Optima 7000 DV, Perkin Elmer, Waltham, MA).

### Sample collection and analytical procedures

Samples of TMR were collected weekly. Samples were dried in a forced-air oven at 70 °C for 48 h for dry matter (DM) determination. Individual steer dry matter intake (**DMI**) was calculated from as-fed intakes (feed disappearance tracked by the unique EID assigned to each steer prior to study initiation) corrected for the DM of weekly TMR samples. Samples of the control treatment TMR were dried, ground, and composited for analysis of nitrogen, neutral detergent fiber, and ether extract by a commercial laboratory (Dairyland Laboratories, Inc., Arcadia, WI). Feed efficiency (G:F) was calculated from the total gain and total DMI during weighing intervals. Dried, ground, and composited TMR were acid digested using TM grade nitric acid as previously described (Genther-Schroeder et al., 2016) before analysis for Cu, Fe, Mn, and Zn concentrations using inductively coupled plasma optical emission spectroscopy (Optima 7000; PerkinElmer, Waltham, MA).

Liver biopsies and muscle biopsies from the longissimus thoracis were collected from all steers (*n* = 144) on days 0, 20, 40, and 84 relative to implant administration using the methods outlined by Engle and Spears (2000) and Pampusch et al. (2003), respectively. A subset of liver samples from 72 steers (12 per treatment) were used for analysis of liver TM concentration. This subset, referenced here and throughout, was selected based on cumulative average daily gain (**ADG**), such that the average of the subset was similar to the average of the full set within a given treatment. An equal number of steers were sampled within respective treatments and block. Liver samples were dried and acid digested in preparation for subsequent analysis of Cu, Fe, Mn, and Zn concentration via ICP-OES using methods previously described (Pogge and Hansen, 2013). A bovine liver reference sample from National Institutes of Standards and Technology (Gaithersburg, MD) was included in all analyses to verify instrument accuracy.

Whole blood samples were collected from all steers (*n* = 144) on days 0, 20, 40, and 84 approximately 2 h postfeeding in concurrence with both liver and muscle biopsies. Whole blood samples (collected in tubes containing either no additive for serum, K_2_EDTA for TM analysis) were centrifuged at 1,000 × *g* for 20 min at 4 °C. Serum was aliquoted and stored at −80 °C while plasma was aliquoted and stored at −20 °C prior to sample analysis.

A subset of steers (*n* = 72; 12 steers per treatment) was selected for analysis of circulating metabolites, as well as plasma and liver TM concentration. Plasma samples were analyzed for Cu, Fe, and Zn concentration within the aforementioned subset of steers methods previously described (Pogge and Hansen, 2013). A human serum reference sample from UTAK Laboratories Inc. (Valencia, CA) was included in all analyses to verify instrument accuracy. Serum glucose, insulin, nonesterified fatty acid (**NEFA**), Urea-N, and IGF-I were determined for these same steers on days 0, 20, 40, and 84 relative to implant using commercially available kits; glucose (FUJIFILM Wako Diagnostics; intra-assay CV = 8.7%, interassay CV = 6.5%), insulin (Bovine Insulin ELISA assay; Mercodia, Inc., Winston Salem, NC; intra-assay CV = 12.1%, interassay CV = 8.9%), NEFA (Wako Pure Chemical Industries Ltd., Chuo-Ku Osaka, Japan; intra-assay CV = 2.1%, interassay CV = 12.9%), serum urea-N (SUN; Teco Diagnostics, Anaheim, CA; intra-assay CV = 4.9%, interassay CV = 6.6%), and serum IGF-I (SG100; R&D Systems, Minneapolis, MN; intra-assay CV = 6.5%, interassay CV = 3.2%). Glucose, NEFA, and insulin concentrations were used to determine the revised quantitative insulin sensitivity check index (RQUICKI) which was calculated using an equation adapted from de Sousa et al. (2022): RQUICKI = 1/[log(Glucose) + log(Insulin) + log(NEFA)] (de Sousa et al., 2022). A greater RQUICKI index value is indicative of greater insulin sensitivity. The RQUICKI index value serves as a proxy for insulin sensitivity in cattle rather than a true insulin tolerance test.

### Isolation of RNA, quantification, and cDNA synthesis

Previously published methods were used to isolate RNA (Reichhardt et al., 2021a) from a subset of steers (*n* = 72; 12 steers per treatment). Flash-frozen skeletal muscle samples were ground under liquid nitrogen by mortar and pestle. Mortar and pestle were autoclaved between samples. Extraction of RNA was performed using TriZol following the manufacturer’s protocol (Invitrogen, Carlsbad, 138 CA). All RNA samples were treated with 144 deoxyribonuclease (Ambion, Foster City, CA) before being converted to cDNA by way of high-capacity cDNA reverse transcription kit (Applied Biosystems, Foster City, CA) following the manufacturer’s protocol.

### Fluidigm reverse transcription qPCR

A total of 45 genes were targeted for analysis of quantitative gene expression in flash-frozen muscle tissue from days 20 and 84 postimplant and were examined on two chips (Supplementary Table S1) via the 96.96 Dynamic Array Integrated Fluidic Circuit (IFC; Fluidigm, San Francisco, CA, USA). This array of target genes in skeletal muscle was selected for their functional relevance in satellite cell function, muscle growth, energy metabolism, mineral homeostasis, and antioxidant capacity. The mRNA abundance of selected genes was used to explore the physiological impacts of Zn on the skeletal muscle of implanted and non-implanted beef steers. Transcript abundances were normalized to the housekeeping gene ribosomal protein S9 (**RPS9**) using the per sample Ct method (Żochowska-Kujawska, 2016). Primers (*n* = 45 total) were designed by Standard Biotools (Standard BioTools, formerly Fluidigm; Supplementary Table S1). In brief, as described by Reichhardt et al. (2021a) following the Fluidigm protocol, a specific target amplification (**STA**) was performed to enrich each sample for target-specific cDNA prior to quantitative PCR. For STA thermal cycling, each reaction consisted of 1.25 μL of primer mix, 2.5 μL of the TaqMan PreAmp Master Mix (Applied Biosystems; Foster City, CA), and 1.25 μL of cDNA. Enzyme activation took place at 95 °C for 10 min and then the amplification took place over 14 cycles (95 °C for 15 s then 60 °C for 4 min). The Fluidigm IFC chip was then run on the Biomark (Fluidigm, San Francisco, CA, USA) thermocycler/detection module. Data for mRNA abundance are presented as ΔCt values adjusted for the endogenous housekeeping gene RPS9. Relative to RPS9, lesser ΔCt values indicate lesser cycle threshold value and thus greater mRNA abundance/gene expression.

### Statistical analysis

Feedlot growth, carcass data, and abundance of muscle mRNA on days 20 and 84 were analyzed by ANOVA as a randomized complete block design using the MIXED procedure of SAS 9.4 (SAS Inst. Inc., Cary, NC). The study employed a factorial design with a split-plot approach. Pens were used as the whole plot units and were assigned to a specific combination of zinc treatment and implant, and individual steer within the pen was the experimental unit. The model included fixed effects of dietary Zn treatment (ZINC), implant (IMP), their interaction, and block. Individual steer within the pen was included in the model as a random effect. For gene expression data and carcass characteristics, no interactions between ZINC and IMP were noted, and thus linear and quadratic contrast statements were constructed for ZINC, as unequally spaced treatments using the IML procedure of SAS 9.4. For IMP, treatment means for carcass characteristics and mRNA abundance data were separated using the LSMEANS statement with the PDIFF option. Growth performance data, plasma and liver TM data, and blood metabolite data were analyzed as repeated measures using the MIXED procedure of SAS 9.4 (SAS Inst. Inc., Cary, NC) with period or day of sampling as the repeated effect. Covariance matrix structures were selected based on the lowest Akaike information criterion. Heterogeneous autoregressive was utilized for liver TM, BW, ADG, G:F, and DMI utilized. An unstructured covariance matrix was utilized for SUN, IGF-1, and glucose data. Autoregressive covariance matrix was utilized for RQUICKI data. Heterogeneous compound symmetry was utilized for plasma TM, NEFA, and insulin data. Initial BW (day −60) served as a covariate in performance data analysis. As no blood or liver sample was collected on day −60 when dietary treatments began, no covariate was included for these measures during subsequent data analysis. Statistical outliers were determined to be data that were beyond three SDs from the mean for a particular parameter. Significance was determined as *P* ≤ 0.05 and tendencies were declared when 0.05 < *P* ≤ 0.10.

## Results

### Growth performance and carcass characteristics

**Figure 1.**
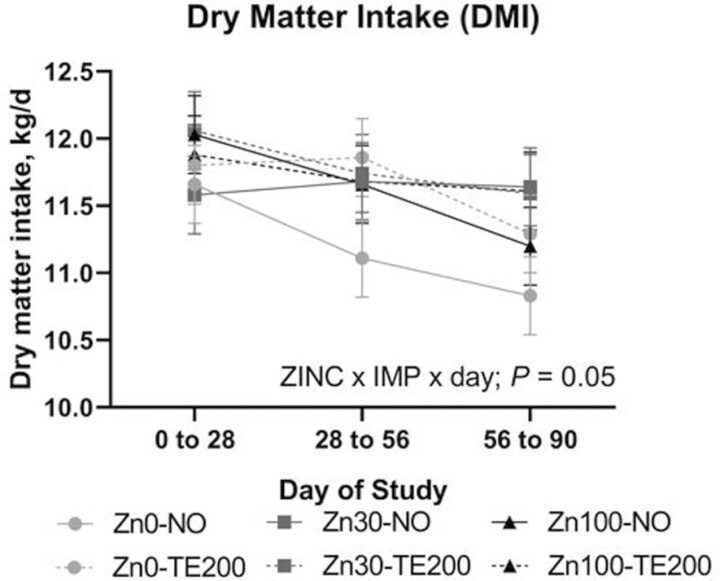
The effect of ZINC × IMP × day (*P* = 0.05) on DMI. Treatments were relatively constant across time and did not differ from one another within a given period, but Zn0-NO and Zn100-NO decreased over the course of the study while other treatments did not decrease to the same degree. Dietary treatments (ZINC; eight pens per treatment) included supplemental Zn as ZnSO_4_ at: 1) 0 (analyzed 54 mg Zn/kg DM; Zn0); 2) 30 mg/kg DM (Zn30); 3) 100 mg Zn/kg DM (Zn100). Steers received a steroidal implant treatment (IMP) on day 0: 1) no implant; NO; or 2) high-potency combination implant (TE-200, Elanco, Greenfield, IN; 200 mg TBA, 20 mg E2; TE200). Data were analyzed as repeated measures of the mixed procedure of SAS; for NO and TE200 *n* = 35 to 36 steers per sampling timepoint.

**Figure 2.**
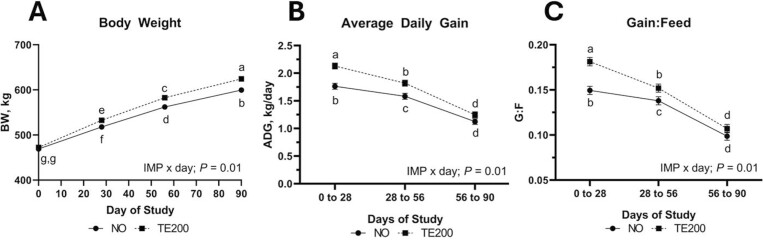
The effect of IMP × day (*P* ≤ 0.01) on BW (A), ADG (B), and G:F (C). Steers received a steroidal implant treatment (IMP) on day 0: 1) no implant; NO; or 2) high-potency combination implant (TE-200, Elanco, Greenfield, IN; 200 mg TBA, 20 mg E2; TE200). Data were analyzed as repeated measures of the mixed procedure of SAS; for NO and TE200 *n* = 35 to 36 steers per sampling timepoint. Within a panel, ^a,b^unlike superscripts differ (*P* ≤ 0.05).

A ZINC × IMP × day effect was noted for DMI (*P *= 0.02) where treatments were relatively constant across time and did not differ from one another within a given interim period, but Zn0-NO and Zn100-NO decreased over the course of the study while other treatments did not decrease to the same degree (Figure 1). No effect of ZINC or ZINC × Day (*P *≥ 0.22) was noted for BW or ADG, but G:F tended to be affected by ZINC × Day (Table 2; *P *= 0.06) driven by Zn30 and Zn100 decreasing from period 0-28 to period 28-56 while Zn0 was similar in both periods. An IMP × Day effect was noted for BW (*P *= 0.01) where BW increased over time and was greatest in TE200 for days 28, 56, and 90 (Figure 2A). An IMP × day effect (*P *≤ 0.01) was noted for ADG and G:F where TE200 was greater during period 0-28 and period 28-56 while NO and TE200 were not different from days 56 to 84 (Figure 2B and C).

**Table 2.** Influence Zn supplementation on growth performance in beef steers^1^

	ZINC^2^			SEM	*P*-value		
	Zn0	Zn30	Zn100		ZINC	Day	ZINC × day
Steers	48	47	48				
BW, kg^3^^,^^4^	543	546	546	2.6	0.78	0.01	0.24
0	469	471	471	—	—	—	—
28	522	526	527	—	—	—	—
56	571	574	572	—	—	—	—
90	610	611	615	—	—	—	—
ADG, kg/d^4^^,^^5^	1.59	1.60	1.65	0.042	0.60	0.01	0.19
Days 0 to 28	1.88	1.97	1.99	—	—	—	—
Days 28 to 56	1.75	1.71	1.65	—	—	—	—
Days 56 to 90	1.15	1.11	1.30	—	—	—	—
G:F^4^	0.139	0.136	0.138	0.0034	0.86	0.01	0.06
Days 0 to 28	0.161^wx^	0.168^w^	0.167^w^	—	—	—	—
Days 28 to 56	0.153^xy^	0.146^y^	0.137^y^	—	—	—	—
Days 56 to 90	0.103^z^	0.095^z^	0.110^z^	—	—	—	—

^1^Day −60 BW (start of ZINC) served as a covariate in analysis.

^2^Dietary treatments (ZINC; eight pens per treatment) included supplemental Zn as ZnSO4 at: 1) 0 (analyzed 54 mg Zn/kg DM; Zn0); 2) 30 mg/kg DM (Zn30); 3) 100 mg Zn/kg DM (Zn100). After 60 d of Zn treatment, steers received a steroidal implant treatment (IMP) on day 0: 1) no implant; NO; or 2) high-potency combination implant (TE-200, Elanco, Greenfield, IN; 200 mg TBA, 20 mg E2; TE200).

^3^Final BW (day 90) is based off of consecutive day (days 89 and 90) BW collected prior to harvest.

^4^LSmeans are based on overall repeated measures analysis from days 0 through 90 of the experiment.

^5^Superscripts (w, x, y, z) represent differences within ZINC × day effect across sampling period.

Steroidal implant administration increased HCW (Table 3; *P* = 0.01), REA (*P* = 0.01), and tended to increase DP (*P* = 0.06), but did not influence marbling score, RF, YG, or EBF (*P* ≥ 0.11). No linear or quadratic effects of Zn were noted for HCW, REA, RF, DP, marbling score, or YG (*P* ≥ 0.15). However, Zn quadratically affected EBF (*P* = 0.05) where Zn30 was lesser than Zn0 and Zn100.

**Table 3.** Zn supplementation and steroidal implants influence on carcass characteristics in beef steers^1^

	ZINC			SEM	Contrasts		IMP		SEM	*P*-value
	Zn0	Zn30	Zn100		L	Q	NO	TE200		
Steers	48	47	48				72	71		
Carcass characteristics										
HCW, kg	388	391	391	3.7	0.52	0.64	381	399	3.0	0.01
REA, cm^2^	93.0	94.0	91.5	0.99	0.16	0.27	91.5	94.1	0.82	0.02
RF, cm	1.41	1.31	1.38	0.049	0.86	0.15	1.37	1.36	0.041	0.79
DP, %	63.5	63.8	63.8	0.20	0.40	0.31	63.5	63.9	0.17	0.06
Marbling^3^	533	512	550	16.4	0.31	0.21	547	516	13.3	0.11
Yield grade^4^	2.79	2.67	2.85	0.080	0.39	0.18	2.76	2.79	0.066	0.79
EBF, %^5^	30.4	29.7	30.6	0.30	0.37	0.05	30.3	30.2	0.24	0.62

^1^Day −60 BW (start of ZINC) served as a covariate in analysis.

^2^Dietary treatments (ZINC; eight pens per treatment) included supplemental Zn as ZnSO4 at: 1) 0 (analyzed 54 mg Zn/kg DM; Zn0); 2) 30 mg/kg DM (Zn30); 3) 100 mg Zn/kg DM (Zn100). After 60 d of Zn treatment, steers received a steroidal implant treatment (IMP) on day 0: 1) no implant; NO; or 2) high-potency combination implant (TE-200, Elanco, Greenfield, IN; 200 mg TBA, 20 mg E2; TE200).

^3^Marbling scores: slight: 300, small: 400, modest: 500.

^4^Yield grade was assigned by the personnel at the commercial abattoir.

^5^Equations from (Guiroy et al., 2002) were used to estimate EBF using carcass measurements including RF thickness, HCW, USDA quality grade, and longissimus muscle area (REA).

HCW, hot carcass weight; REA, ribeye area; RF, 12th rib fat; DP, dressing percentage; EBF, empty body fat percentage (EBF).

### Plasma TM concentrations

**Figure 3.**
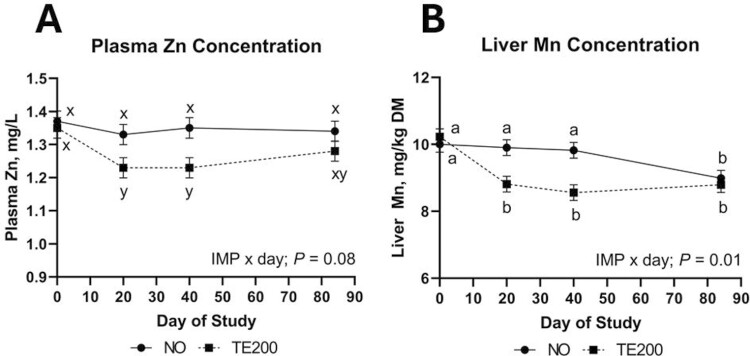
The effect of IMP × day on plasma Zn concentration (*P* = 0.08; A) and liver Mn concentration (*P* = 0.01; B). Steers received a steroidal implant treatment (IMP) on day 0: 1) no implant; NO; or 2) high-potency combination implant (TE-200, Elanco, Greenfield, IN; 200 mg TBA, 20 mg E2; TE200). Data were analyzed as repeated measures of the mixed procedure of SAS; for NO and TE200 *n* = 35 to 36 steers per sampling timepoint. Within a panel, ^a,b^unlike superscripts differ (*P* ≤ 0.05); ^x,y^unlike superscripts tend to differ (0.05 < *P* ≤ 0.10).

**Figure 4.**
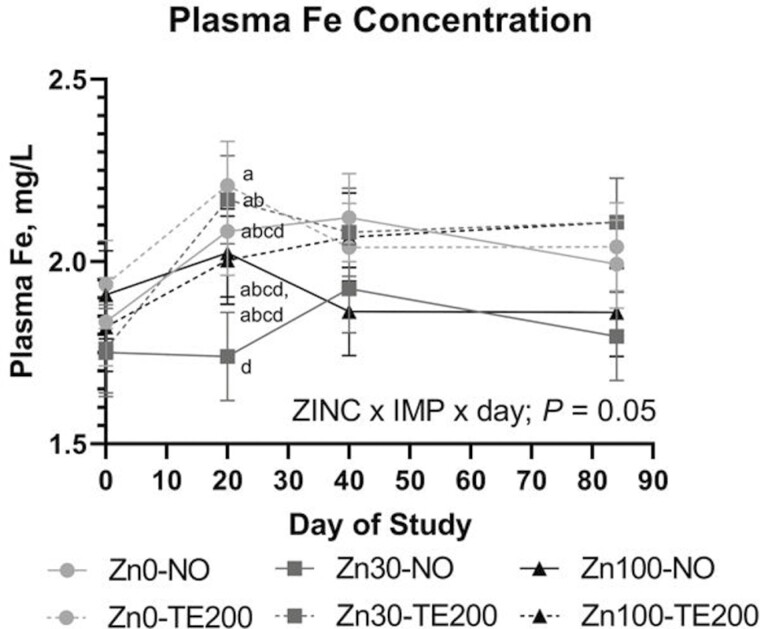
The effect of ZINC × IMP × day (*P* = 0.05) on plasma Fe concentration. Dietary treatments (ZINC; eight pens per treatment) included supplemental Zn as ZnSO_4_ at: 1) 0 (analyzed 54 mg Zn/kg DM; Zn0); 2) 30 mg/kg DM (Zn30); 3) 100 mg Zn/kg DM (Zn100). Steers received a steroidal implant treatment (IMP) on day 0: 1) no implant; NO; or 2) high-potency combination implant (TE-200, Elanco, Greenfield, IN; 200 mg TBA, 20 mg E2; TE200). Data were analyzed as repeated measures of the mixed procedure of SAS; for NO and TE200 *n* = 35 to 36 steers per sampling timepoint. Within day, ^a,b^unlike superscripts differ (*P* ≤ 0.05).

No ZINC, ZINC × day, or ZINC × IMP effects were noted for plasma Cu, Fe, or Zn (*P *≥ 0.26). Plasma Zn and Cu concentrations were not affected by ZINC × IMP × day (*P *≥ 0.15). After 60 d of dietary Zn treatments, plasma Zn concentration averaged 1.36 mg/L and did not differ within ZINC (*P *= 0.26). There was a tendency for an IMP × day effect (*P *= 0.08) on plasma Zn where from days 0 to 20 plasma Zn concentration of TE200 decreased 8.4% while NO cattle decreased only 3.6% (Figure 3A). This relationship held through day 40 where TE200 had lesser plasma Zn concentration compared to NO. Plasma Cu was not influenced by IMP, or IMP × day (*P *≥ 0.19) but increased over the duration of the study (day; *P *= 0.01) where concentrations were 0.79, 0.83, 0.84, and 0.86 (SEM = 0.012) mg Cu/L on days 0, 20, 40, and 84, respectively. A ZINC × IMP × day effect was noted for plasma Fe (*P *≥ 0.05) driven primarily by variation in plasma Fe concentration at days 20 and 40 where Zn30-NO had the lowest plasma Fe concentration at day 20 (Figure 4).

### Liver TM concentrations

Liver TM concentration was not affected by ZINC × IMP × day, ZINC × IMP, or ZINC × day (*P *≥ 0.17). Liver Zn concentration was not affected by ZINC, IMP, day, or any interaction (*P *≥ 0.16; Table 4). Liver Mn was unaffected by ZINC (*P *= 0.75), but liver Mn (Figure 3A) decreased 13.8% from days 0 to 20 in TE200 while NO decreased 1.2% (IMP × day; *P* = 0.01). This relationship where TE200 had lesser liver Mn compared to NO persisted through day 40 but by day 84 liver Mn concentrations did not differ by IMP treatment. Liver Cu was not affected by IMP, ZINC × day, ZINC × IMP, or ZINC × IMP × day (*P *≥ 0.23) but steers fed Zn0 had greater liver Cu concentration than Zn30 or Zn100 (ZINC; *P *= 0.01). Liver Cu increased over the duration of the study (day; *P *= 0.01). Liver Fe was not affected by ZINC, IMP, IMP × day (*P *≥ 0.13) but differed over time where it was lowest on day 0, intermediate on days 20 and 40, and greatest by day 84 (day; *P *= 0.01).

**Table 4.** Zn supplementation and steroidal implants influence on liver TM concentration in beef steers^1^

	ZINC^2^			SEM	IMP^2^		SEM	*P*-value				
	Zn0	Zn30	Zn100		NO	TE200		ZINC	IMP	Day	ZINC × day	IMP × day
Steers	48	47	48		35	36						
Liver Cu, mg/kg DM	337^a^	276^b^	275^b^	15.3	291	301	12.8	0.02	0.59	0.01	0.23	0.55
Day 0	309	253	231	—	264	265	—	—	—	—	—	—
Day 20	334	271	265	—	288	292	—	—	—	—	—	—
Day 40	351	286	289	—	300	317	—	—	—	—	—	—
Day 84	355	295	317	—	314	330	—	—	—	—	—	—
Liver Fe, mg/kg DM	154	157	155	3.6	157	154	2.8	0.77	0.48	0.01	0.91	0.12
Day 0	151	151	146	—	147	152	—	—	—	—	—	—
Day 28	149	154	153	—	156	148	—	—	—	—	—	—
Day 56	153	160	158	—	161	153	—	—	—	—	—	—
Day 90	163	165	161	—	162	163	—	—	—	—	—	—
Liver Mn, mg/kg DM	9.2	9.5	9.4	0.24	9.7	9.1	0.20	0.75	0.06	0.01	0.71	0.01
Day 0	10.0	10.1	10.2	—	10.0^a^	10.2^a^	—	—	—	—	—	—
Day 28	9.2	9.5	9.3	—	9.9^a^	8.8^b^	—	—	—	—	—	—
Day 56	9.1	9.5	9.0	—	9.8^a^	8.6^b^	—	—	—	—	—	—
Day 90	8.7	8.9	9.1	—	9.0^b^	8.8^b^	—	—	—	—	—	—
Liver Zn, mg/kg DM	122	130	131	3.9	127	128	3.0	0.16	0.75	0.27	0.95	0.54
Day 0	121	126	128	—	126	125	—	—	—	—	—	—
Day 28	119	131	129	—	127	126	—	—	—	—	—	—
Day 56	122	131	132	—	128	129	—	—	—	—	—	—
Day 90	125	133	135	—	128	134	—	—	—	—	—	—

^1^Day −60 BW (start of ZINC) served as a covariate in analysis.

^2^Dietary treatments (ZINC; eight pens per treatment) included supplemental Zn as ZnSO4 at 1) 0 (analyzed 54 mg Zn/kg DM; Zn0); 2) 30 mg/kg DM (Zn30); 3) 100 mg Zn/kg DM (Zn100). After 60 d of Zn treatment, steers received a steroidal implant treatment (IMP) on day 0: 1) no implant; NO; or 2) high-potency combination implant (TE-200, Elanco, Greenfield, IN; 200 mg TBA, 20 mg E2; TE200).

^3^Final BW (day 90) is based off of consecutive day (days 89 and 90) BW collected prior to harvest.

^4^LSmeans are based on overall repeated measures analysis from days 0 through 90 of the experiment. ^5^Superscripts (a,b,c) represent differences within the ZINC × day effect across sampling period.

^5^There were no ZINC × IMP × day effects (*P* ≥ 0.18) was not significant for any liver Cu, Fe, Mn, or Zn.

### Serum metabolites and insulin sensitivity (RQUICKI)

**Figure 5.**
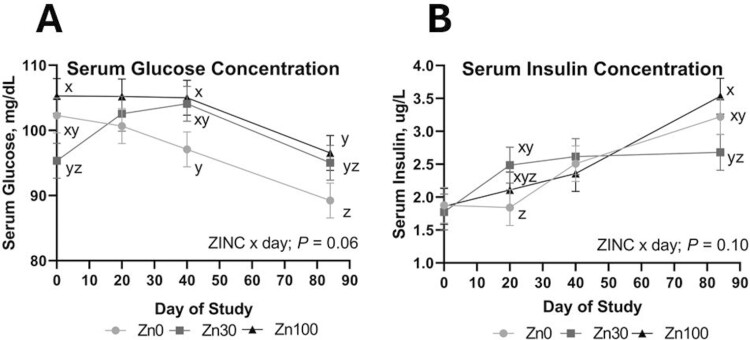
The effect of ZINC × day on serum glucose (*P* = 0.06; A) and insulin (*P* = 0.10; B) concentrations. Dietary treatments (ZINC; eight pens per treatment) included supplemental Zn as ZnSO_4_ at: 1) 0 (analyzed 54 mg Zn/kg DM; Zn0); 2) 30 mg/kg DM (Zn30); 3) 100 mg Zn/kg DM (Zn100). Data were analyzed as repeated measures of the mixed procedure of SAS; for Zn0, Zn30, and Zn100 *n* = 21 to 24 steers per sampling timepoint. Within a panel ^x,y^unlike superscripts tend to differ (0.05 < *P* ≤ 0.10).

**Figure 6.**
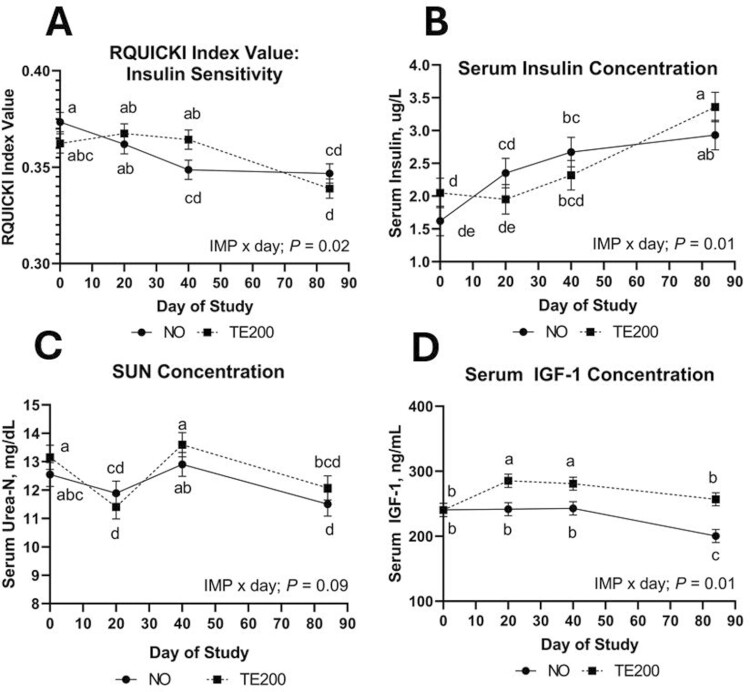
The effect of IMP × day on serum insulin concentration (*P* = 0.02; A), RQUICKI index value (*P* = 0.01; B), SUN (*P* = 0.09; C) and IGF-1 concentration (*P* = 0.01; D). RQUICKI = 1/[log(Glucose) + log(Insulin) + log(NEFA)]. Steers received a steroidal implant treatment (IMP) on day 0: 1) no implant; NO; or 2) high-potency combination implant (TE-200, Elanco, Greenfield, IN; 200 mg TBA, 20 mg E2; TE200). Data were analyzed as repeated measures of the mixed procedure of SAS; for NO and TE200 *n* = 31 to 36 steers per sampling timepoint. Within a panel, ^a,b^unlike superscripts differ (*P* ≤ 0.05).

No ZINC or ZINC × IMP × day effects were noted (*P *≥ 0.12) for any serum metabolites or RQUICKI index value. Serum insulin was not affected by IMP or ZINC × IMP (*P *≥ 0.26) but tended to be affected by ZINC × day (*P* = 0.10), where Zn30 increased from days 0 to 20 and remained similar through day 84 while Zn0 and Zn100 increased over time (Figure 5B). Additionally, serum insulin increased 45% in NO steers from days 0 to 40 while serum insulin concentration for TE200 steers did not change in that time (IMP × day; *P* = 0.01; Figure 6B). Serum glucose was not affected by IMP, IMP × day, or ZINC × IMP × day (*P *≥ 0.23) but was affected by ZINC × day (*P* = 0.07; Figure 5A) where day 0 serum glucose concentration was greatest in Zn100 compared to Zn30 with Zn0 intermediate. Following differences in serum glucose on day 0, this interaction is driven primarily by Zn30 serum glucose increasing through day 40, while Zn0 decreased steadily across DOF. Serum NEFA concentration was not affected by IMP or ZINC × day (*P *≥ 0.58) but changed over time where NEFA concentration decreased from days 0 to 20 and increased through day 84 (day; *P *= 0.01). Concentrations were 155, 127, 128, and 137 (SEM = 4.1) meq/L on sampling days 0, 20, 40, and 84, respectively. RQUICKI was not affected by IMP, ZINC × day, or ZINC × IMP (*P *≥ 0.20). An IMP × day effect (*P* = 0.03) was noted for RQUICKI index value where TE200 was greater on day 40 compared to NO cattle, but by day 84 RQUICKI index value was not different between TE200 and NO (Figure 6A). SUN was not influenced by IMP, ZINC × day, or ZINC × IMP (*P *≥ 0.25). SUN (Figure 6C) tended to differ as a result of implant over time (IMP × day; *P* = 0.09). This tendency was driven by TE200 decreasing from day 0 to 20, however, within any given day TE200 and NO were not different. Serum IGF-1 was not affected by ZINC × day or ZINC × IMP (*P *≥ 0.13). An IMP × day effect was noted (*P *= 0.04) for circulating IGF-1 where NO and TE200 were similar on the day of implant (day 0); however, from days 20 through 84, serum IGF-1 was greater in TE200 compared to NO (Figure 6D).

### Abundance of mRNA in the longissimus thoracis 20 and 84 d postimplant

#### Abundance of mRNA for genes associated with muscle growth and energy metabolism

Genes associated with muscle growth and energy metabolism on days 20 and 84 are displayed in Tables 5 and 6, respectively. Data are presented as ΔCt values where lesser values indicate lesser cycle threshold value and thus greater mRNA abundance/gene expression. Tendencies for negative linear effects of ZINC were noted for day 20 on protein kinase B (AKT1), mammalian target of rapamycin (mTOR), and muscle RING finger 1 (TRIM63; *P* ≤ 0.09) where mRNA abundance increased with increasing Zn supplementation. On day 84, a tendency (*P *= 0.08) for a quadratic response to ZINC was noted where AKT1 mRNA abundance was lowest in Zn30 while no linear or quadratic effects were noted for mTOR or TRIM63 on day 84 (*P *≥* *0.12). A negative linear response was noted for day 20 matrix metalloproteinase 2 (MMP2) and day 20 myogenic regulatory factor 5 (MYF5), where mRNA abundance again increased with increasing Zn supplementation (*P* ≤ 0.03), but no linear or quadratic effect of ZINC were noted on day 84 (*P *≥* *0.14). Similar quadratic tendencies were noted (*P* = 0.07) for both 20 and 84 secreted protein acidic and cysteine rich (SPARC) where mRNA abundance was lowest in Zn30. On day 84, a tendency for a positive linear effect of ZINC was noted for both insulin-like growth factor 1 receptor (IGF1R) and myogenin differentiation factor 1 (MYOD1), where mRNA abundance was greatest in Zn0, no linear or quadratic effects of ZINC were noted for IGF1R and MYOD1 on day 20 (*P *≥* *0.11). On day 84 a negative quadratic effect was noted for myogenin (MYOG) where mRNA abundance is greater in Zn0 and lesser in Zn30 and Zn100. No linear or quadratic effects of ZINC were noted for MYOG on day 20 (*P *≥* *0.18). Two genes were influenced by IMP treatment on day 20, MY5 and solute carrier family 2 member 4 (SLC2A4) also known as glucose transporter 4 (**GLUT4**). Abundance of day 20 MYF5 mRNA tended to be greater (*P* = 0.10) in TE200 than NO while day 20 SLC2A4 mRNA abundance tended to be greater in NO (*P* = 0.10). No other genes on either days 20 or 84 in this category were influenced by IMP (*P *≥* *0.13).

**Table 5.** Day 20 abundance of mRNA for genes associated with muscle growth and energy metabolism (presented as ΔCt values) in skeletal muscle of beef steers given a steroidal implant and supplemented differing concentrations of Zn^1^^,^^2^

	ZINC			SEM	Contrasts		IMP		SEM	*P*-value
	Zn0	Zn30	Zn100		L	Q	NO	TE200		
N	10	14	10				20	14		
GENE3										
AKT1	2.44	2.42	2.04	0.182	0.09	0.61	2.27	2.33	0.153	0.77
CAPN6	5.97	6.00	6.10	0.331	0.77	0.99	6.04	6.00	0.267	0.91
CKM	−4.60	−4.69	−4.62	0.119	0.99	0.55	−4.63	−4.64	0.098	0.92
CKMT2	−1.36	−1.23	−1.47	0.183	0.55	0.41	−1.28	−1.44	0.154	0.42
CS	0.49	0.66	0.17	0.173	0.14	0.21	0.48	0.40	0.159	0.70
DCN	2.63	2.99	2.26	0.460	0.44	0.40	2.76	2.50	0.379	0.61
FNDC5	3.81	4.05	3.71	0.189	0.52	0.26	3.91	3.80	0.159	0.62
FOXO4	3.57	3.56	3.23	0.244	0.28	0.72	3.58	3.34	0.205	0.36
GLUD1	2.26	2.27	1.95	0.210	0.23	0.64	2.18	2.14	0.176	0.87
IGF1R	6.12	5.66	5.59	0.254	0.21	0.36	5.73	5.85	0.213	0.70
IR	4.34	4.46	4.14	0.170	0.30	0.34	4.32	4.03	0.143	0.92
LDHB	1.13	1.52	0.98	0.271	0.47	0.13	1.27	1.14	0.219	0.64
MEF2C	1.53	1.42	1.26	0.179	0.28	0.87	1.41	1.40	0.150	0.94
MHCI	−4.95	−4.80	−5.07	0.150	0.44	0.26	−5.02	−4.85	0.127	0.30
MHCIIx	−4.37	−5.01	−5.47	0.485	0.13	0.58	−5.03	−4.87	0.386	0.76
MMP2	4.30	4.33	3.71	0.211	0.03	0.36	3.94	4.25	0.177	0.25
MT2A	3.98	4.34	4.12	0.412	0.93	0.54	4.14	4.15	0.369	0.99
mTOR	3.97	4.22	3.50	0.222	0.07	0.11	3.80	4.00	0.187	0.42
MYF5	7.32	7.23	6.22	0.310	0.02	0.50	7.23	6.62	0.208	0.10
MYF6	1.49	1.84	1.38	0.318	0.62	0.31	1.59	1.54	0.254	0.88
MYH2	−3.81	−3.83	−3.77	0.223	0.86	0.90	−3.75	−3.85	0.187	0.67
MYOD1	2.56	3.32	2.93	0.333	0.64	0.11	3.07	2.80	0.263	0.45
MYOG	2.90	3.65	3.15	0.394	0.89	0.18	3.28	3.18	0.314	0.82
PAX7	4.53	4.69	4.50	0.266	0.84	0.62	4.45	4.70	0.233	0.43
PDE4B	4.49	4.49	3.93	0.391	0.27	0.72	4.40	4.21	0.319	0.66
PDP1	2.65	2.63	2.18	0.239	0.13	0.64	2.62	2.36	0.201	0.32
PRKAA2	1.00	1.43	1.10	0.250	0.99	0.20	1.26	1.10	0.203	0.57
PYGM	−3.77	−3.66	−3.83	0.145	0.61	0.41	−3.87	−3.63	0.122	0.13
RHEB	1.61	1.85	1.09	0.557	0.41	0.55	1.72	1.31	0.449	0.51
SDHA	0.40	0.66	0.07	0.263	0.24	0.19	0.36	0.39	0.213	0.91
SLC2A4	1.69	1.90	1.63	0.141	0.54	0.13	1.61	1.87	0.118	0.10
SPARC	1.52	2.60	1.18	0.547	0.40	0.07	1.34	2.20	0.466	0.16
TRIM63	1.56	1.19	0.82	0.205	0.03	0.55	1.31	1.08	0.182	0.34

^1^Dietary treatments (ZINC; eight pens per treatment) included supplemental Zn as ZnSO_4_ at 1) 0 (analyzed 54 mg Zn/kg DM; Zn0); 2) 30 mg/kg DM (Zn30); 3) 100 mg Zn/kg DM (Zn100). After 60 d of Zn treatment, steers received a steroidal implant treatment (IMP) on day 0: 1) no implant; NO; or 2) high-potency combination implant (TE-200, Elanco, Greenfield, IN; 200 mg TBA, 20 mg E2; TE200).

^2^All ΔCt values are adjusted for the housekeeping gene (RPS9). Relative to RPS9, lesser ΔCt values indicate lesser cycle threshold value and thus greater mRNA abundance/gene expression.

^3^AKT1, protein kinase B; CAPN6, calpain 6; CKM, creatine kinase, M-type; CKMT2, creatine kinase, mitochondrial 2; CS, citrate synthase; DCN, decorin; FNDC5, fibronectin type III domain containing 5; FOXO4, forkhead box 04; GLUD1, glutamate dehydrogenase 1; IGF1R, insulin-like growth factor 1 receptor; IR, insulin receptor; LDHB, lactate dehydrogenase B; MEF2C, myocyte enhancer factor 2C; MHCI, myosin heavy chain I; MHCIIx, myosin heavy chain IIx; MMP2, matrix metalloproteinase 2; MT2A, metallothionein 2A; mTOR, mammalian target of rapamycin; MYF5, myogenic regulatory factor 5; MYF6, myogenic regulatory factor 6; MYH2, myosin heavy chain 2; MYOD1, myogenin differentiation factor 1; MYOG, myogenin, paired box protein PAX7; PDE4B, phosphodiesterase 4B; PDP1, pyruvate dehydrogenase phosphatase; PRKAA2, protein kinase AMP-activated catalytic subunit alpha 2; PYGM, glycogen phosphorylase; RHEB, Ras homolog, mTORC1 binding; SDHA, succinate dehydrogenase; SLC2A4, solute carrier family 2 member 4 (GLUT4); SPARC, secreted protein acidic and cysteine rich; TRIM63, muscle RING finger 1 (MuRF1).

**Table 6.** Day 84 abundance of mRNA for genes associated with muscle growth and energy metabolism (presented as ΔCt values) in skeletal muscle of beef steers given a steroidal implant and supplemented differing concentrations of Zn^1^^,^^2^

	ZINC			SEM	Contrasts		IMP		SEM	*P*-value
	Zn0	Zn30	Zn100		L	Q	NO	TE200		
*n*	17	15	18				25	25		
GENE3										
AKT1	1.13	2.06	1.77	0.286	0.28	0.08	1.65	1.66	0.244	0.99
CAPN6	3.79	5.07	5.01	0.603	0.27	0.30	4.46	4.79	0.538	0.66
CKM	−3.87	−4.29	−4.46	0.335	0.27	0.59	−4.22	−4.19	0.285	0.94
CKMT2	−1.07	−1.27	−1.30	0.156	0.40	0.56	−1.25	−1.17	0.133	0.66
CS	0.20	0.41	0.28	0.150	0.90	0.40	0.26	0.33	0.129	0.73
DCN	1.57	2.05	1.79	0.336	0.81	0.39	1.67	1.94	0.286	0.51
FNDC5	2.62	3.52	3.50	0.397	0.20	0.24	3.20	3.23	0.338	0.96
FOXO4	2.01	2.94	2.74	0.341	0.27	0.15	2.53	2.60	0.290	0.86
GLUD1	1.21	1.81	1.73	0.251	0.27	0.22	1.57	1.60	0.215	0.93
IGF1R	3.59	5.04	5.24	0.548	0.07	0.20	4.73	4.51	0.456	0.75
IR	2.24	3.53	3.71	0.521	0.11	0.25	3.16	3.16	0.444	0.99
LDHB	0.46	0.87	0.66	0.155	0.63	0.11	0.53	0.79	0.132	0.18
MEF2C	0.83	1.04	1.07	0.178	0.42	0.58	1.04	0.92	0.155	0.61
MHCI	−4.11	−4.61	−4.86	0.323	0.16	0.54	−4.56	−4.49	0.275	0.87
MHCIIx	−4.37	−4.41	−5.01	0.455	0.31	0.81	−4.60	−4.60	0.388	0.99
MMP2	2.21	3.38	3.29	0.419	0.15	0.14	2.91	3.01	0.358	0.85
MT2A	2.38	3.27	3.23	0.466	0.28	0.29	3.01	2.91	0.365	0.84
mTOR	2.21	3.20	3.17	0.416	0.21	0.25	2.82	2.89	0.354	0.90
MYF5	4.63	6.13	6.16	0.694	0.22	0.31	5.74	5.53	0.597	0.81
MYF6	0.66	1.14	1.14	0.209	0.25	0.25	1.02	0.92	0.182	0.69
MYH2	−3.06	−3.41	−3.91	0.372	0.11	0.85	−3.46	−3.46	0.292	0.99
MYOD1	1.32	2.28	2.33	0.326	0.09	0.16	2.05	1.91	0.278	0.74
MYOG	1.60	2.73	2.47	0.320	0.16	0.05	2.30	2.24	0.273	0.87
PAX7	2.22	3.52	3.36	0.417	0.16	0.12	3.08	2.99	0.354	0.86
PDE4B	2.73	3.61	3.44	0.469	0.36	0.26	3.33	3.19	0.365	0.78
PDP1	1.33	1.84	2.04	0.314	0.18	0.50	1.72	1.76	0.269	0.93
PRKAA2	0.58	1.01	1.10	0.203	0.15	0.35	0.87	0.92	0.173	0.85
PYGM	−3.18	−3.28	−3.72	0.357	0.25	0.89	−3.38	−3.41	0.280	0.94
RHEB	0.40	0.96	0.78	0.336	0.59	0.36	0.65	0.78	0.288	0.76
SDHA	−0.15	0.20	−0.18	0.172	0.59	0.12	−0.04	−0.04	0.133	0.99
SLC2A4	0.72	1.37	1.23	0.210	0.16	0.06	1.10	1.12	0.165	0.95
SPARC	0.41	1.27	0.97	0.294	0.36	0.09	0.85	0.91	0.251	0.85
TRIM63	0.64	1.02	1.12	0.178	0.12	0.35	0.89	0.96	0.155	0.78

^1^Dietary treatments (ZINC; eight pens per treatment) included supplemental Zn as ZnSO_4_ at 1) 0 (analyzed 54 mg Zn/kg DM; Zn0); 2) 30 mg/kg DM (Zn30); 3) 100 mg Zn/kg DM (Zn100). After 60 d of Zn treatment, steers received a steroidal implant treatment (IMP) on day 0: 1) no implant; NO; or 2) high-potency combination implant (TE-200, Elanco, Greenfield, IN; 200 mg TBA, 20 mg E2; TE200).

^2^All ΔCt values are adjusted for the housekeeping gene (RPS9). Relative to RPS9, lesser ΔCt values indicate lesser cycle threshold value and thus greater mRNA abundance/gene expression.

^3^AKT1, protein kinase B; CAPN6, calpain 6; CKM, creatine kinase, M-type; CKMT2, creatine kinase; mitochondrial 2; CS, citrate synthase; DCN, decorin; FNDC5, fibronectin type III domain containing 5; FOXO4, forkhead box 04; GLUD1, glutamate dehydrogenase 1; IGF1R, insulin-like growth factor 1 receptor; IR, insulin receptor; LDHB, lactate dehydrogenase B; MEF2C, myocyte enhancer factor 2C; MHCI, myosin heavy chain I; MHCIIx, myosin heavy chain IIx; MMP2, matrix metalloproteinase 2; MT2A, metallothionein 2A; mTOR, mammalian target of rapamycin; MYF5, myogenic regulatory factor 5; MYF6, myogenic regulatory factor 6; MYH2, myosin heavy chain 2; MYOD1, myogenin differentiation factor 1; MYOG, myogenin, paired box protein PAX7; PDE4B, phosphodiesterase 4B; PDP1, pyruvate dehydrogenase phosphatase; PRKAA2, protein kinase AMP-activated catalytic subunit alpha 2; PYGM, glycogen phosphorylase; RHEB, Ras homolog, mTORC1 binding; SDHA, succinate dehydrogenase; SLC2A4, solute carrier family 2 member 4 (GLUT4); SPARC, secreted protein acidic and cysteine rich; TRIM63, muscle RING finger 1 (MuRF1).

#### Abundance of mRNA for genes associated with mineral homeostasis and antioxidant capacity

Genes associated with mineral homeostasis and antioxidant capacity on days 20 and 84 are displayed in Table 7.

**Table 7.** Days 20 and 84 abundance of mRNA for genes associated with mineral homeostasis and antioxidant capacity (presented as ΔCt values) in skeletal muscle of beef steers given a steroidal implant and supplemented differing concentrations of Zn^1^^,^^2^

	ZINC			SEM	Contrasts		IMP		SEM	*P*-value
	Zn0	Zn30	Zn100		L	Q	NO	TE200		
n	10	14	10				20	14		
GENE^3^										
Day 20										
GPX1	1.89	2.71	1.29	0.518	0.23	0.12	1.85	2.08	0.418	0.68
NFE2L2	3.57	4.36	2.74	0.830	0.33	0.29	4.03	3.08	0.665	0.31
NFKB2	4.19	4.26	3.77	0.195	0.08	0.37	4.13	4.01	0.164	0.58
PARK7	1.01	1.00	0.59	0.424	0.43	0.79	1.04	0.69	0.343	0.44
RYR1	−1.35	−1.76	−1.80	0.219	0.22	0.31	−1.68	−1.59	0.175	0.70
SLC30A5	5.82	5.89	5.21	0.296	0.11	0.47	5.80	5.48	0.247	0.34
SLC30A7	5.54	5.74	4.74	0.278	0.03	0.20	5.24	5.44	0.235	0.51
SLC39A14	5.78	5.77	5.44	0.310	0.39	0.79	5.49	5.83	0.270	0.33
SLC39A7	5.20	5.47	4.89	0.376	0.44	0.42	5.47	4.91	0.315	0.19
SLC39A8	6.70	7.01	6.65	0.292	0.74	0.30	6.78	6.79	0.187	0.99
Day 84										
GPX1	0.42	1.35	0.65	0.348	0.98	0.07	0.81	0.80	0.268	0.97
NFE2L2	1.69	3.04	2.62	0.618	0.42	0.18	2.33	2.57	0.485	0.72
NFKB2	2.28	3.37	3.32	0.429	0.13	0.15	2.95	3.03	0.333	0.87
PARK7	0.36	0.52	0.29	0.226	0.70	0.52	0.36	0.43	0.176	0.78
RYR1	−1.58	−1.66	−1.77	0.183	0.51	0.94	−1.68	−1.66	0.156	0.91
SLC30A5	4.08	4.99	5.20	0.628	0.25	0.48	4.87	4.64	0.505	0.75
SLC30A7	3.22	4.79	4.76	0.658	0.15	0.19	4.17	4.34	0.512	0.81
SLC39A14	2.99	4.50	4.57	0.694	0.15	0.25	4.07	3.97	0.540	0.90
SLC39A7	2.94	4.57	4.39	0.697	0.21	0.19	3.94	3.99	0.542	0.94
SLC39A8	4.03	5.89	5.70	0.856	0.25	0.22	5.09	5.33	0.672	0.80

^1^Dietary treatments (ZINC; eight pens per treatment) included supplemental Zn as ZnSO_4_ at 1) 0 (analyzed 54 mg Zn/kg DM; Zn0); 2) 30 mg/kg DM (Zn30); 3) 100 mg Zn/kg DM (Zn100). After 60 d of Zn treatment, steers received a steroidal implant treatment (IMP) on day 0: 1) no implant; NO; or 2) high-potency combination implant (TE-200, Elanco, Greenfield, IN; 200 mg TBA, 20 mg E2; TE200).

^2^All ΔCt values are adjusted for the housekeeping gene (RPS9). Relative to RPS9, lesser ΔCt values indicate lesser cycle threshold value and thus greater mRNA abundance/gene expression.

^3^GPX1, glutathione peroxidase 1; NFE2L2, nuclear factor erythroid 2-related factor 2; NFKB2, nuclear factor kappa B subunit 2; PARK7, Parkinsonism associated deglycase; RYR1, ryanodine receptor 1; SLC30A5, solute carrier family 30 member 5 (ZnT5); SLC30A7, solute carrier family 30 member 7 (ZnT7); SLC39A14, solute carrier family 39 member 14 (ZIP14); SLC39A7, solute carrier family 39 member 7 (ZIP7); SLC39A8, solute carrier family 39 member 8 (ZIP8).

Steroidal implant administration did not influence any genes related to mineral homeostasis and antioxidant capacity on day 20 or 84 (*P *≥* *0.19). However, on day 20, a negative linear effect (*P* = 0.03) and a tendency for a negative linear effect of ZINC (*P* = 0.08) were noted for solute carrier family 30 member 7 (SLC30A7) and nuclear factor kappa B subunit 2 (NFKB2), respectively, where mRNA abundance was greatest in the Zn100. No linear or quadratic effects of ZINC were noted for SLC30A7 or NFKB2 (*P *≥* *0.13) on day 84. On day 84, a tendency for a quadratic effect (*P* = 0.07) was noted for glutathione peroxidase 1 (**GPX1**) where mRNA abundance was lowest in Zn30.

## Discussion

This study aimed to determine how increasing concentrations of supplemental Zn affect steroidal implant-induced growth, carcass characteristics, TM status, circulating metabolites, and skeletal muscle gene expression in beef steers. Dietary Zn treatments began 60 d prior to implant administration, while in our previous studies, dietary treatments began concurrent with implant. After 60 d on Zn treatments preimplant, plasma Zn concentrations in steers (1.36 mg/L) were at the upper end of the “adequate” range (Kincaid, 1999). As a result, regardless of ZINC treatment, all steers had excellent Zn status on implant day.

Previous work showed that supplementing up to 150 mg Zn/kg DM from ZnSO_4_ improved growth performance during peak implant payout (Messersmith and Hansen, 2021a; Messersmith et al., 2022). Based on repeated measures, ZINC did not affect BW or ADG, but did differentially influence G:F where this measure decreased over time. The decrease in feed efficiency for Zn100 was less abrupt than Zn0 and Zn30. Unlike prior work (Messersmith et al., 2022) increased Zn supplementation did not improve HCW. In the present study, basal diet Zn concentration was 53 mg Zn/kg DM, nearly double the NASEM recommendation of 30 mg Zn/kg DM for growing and finishing cattle (NASEM, 2016). This, in combination with the 60-d supplementation of Zn prior to implant, may explain why in contrast to other studies, implanted steers did not respond to supplementation Zn in this study.

Similar to our prior observation that implant administration decreases plasma Zn concentrations (Messersmith, 2018; Reichhardt et al., 2022), implant tended to decrease plasma Zn concentrations. Plasma Zn of implanted steers decreased 9.0% from days 0 to 20. The decrease in plasma Zn concentrations reported herein occurred concurrent with the greatest degree of steroidal hormone payout and consequently the period of greatest growth rate. Neither IMP nor increased Zn supplementation influenced liver Zn concentration, as also noted by others (Messersmith and Hansen, 2021b; Messersmith et al., 2021). However, liver Mn decreased in implanted steers by 13.8% from days 0 to 20, consistent with prior reports (Messersmith, 2018; Niedermayer et al., 2018; Reichhardt et al., 2021a; Messersmith et al., 2022).

Data from this experiment indicate implant-induced growth was declining toward the end of the experiment. ADG and G:F were similar between IMP and NO from days 56 to 90 period. Likewise, both liver Mn and plasma Zn were similar between IMP and NO on day 84. Ears were palpated and implant sites were assessed during interim weight measurements to ensure proper implant placement and lack of ear abscesses. Thus, we did not expect this degree of decrease in performance when the terminal implant period was only 90 d. However, serum IGF-1 was greater in TE200 compared to NO from days 20 through 84. This agrees with prior reports (Parr et al., 2014; Smith et al., 2018). Interestingly, gene expression data from this study found no influence of IMP on muscle IGF1R mRNA abundance on day 20 or 84. On day 84, mRNA abundance for IGF1R decreased with increasing ZnSO_4_. Zinc supplementation has been linked to greater circulating concentrations of IGF-1 (Nishi et al., 1989; Ninh et al., 1996; Hamza et al., 2012).

Implanted cattle maintained greater insulin sensitivity longer into the finishing period compared to non-implanted steers. The RQUICKI index value was used as a proxy for insulin sensitivity in this study (de Sousa et al., 2022). Insulin resistance describes a state where the effect of a given concentration of insulin is reduced (Wallace and Matthews, 2002); this may become more important in feedlot cattle as cattle are harvested at increasingly greater fatness (Crawford et al., 2022; Galyean et al., 2023). Insulin-dependent uptake of glucose into skeletal muscle is vital in supporting normal skeletal muscle growth (Petersen and Shulman, 2018). The observed influence of steroidal implant on RQUICKI index value over time suggests this may be a promising area in need of further exploration in finishing cattle. Zinc supplementation resulted in a quadratic effect on EBF but did not influence insulin sensitivity based on RQUICKI index values. This was contrary to our hypothesis in which we expected to see an effect of Zn on insulin sensitivity and may be due to the high basal concentration of dietary Zn.

Insulin is a key mediator of energy metabolism related to nutrient uptake. Zinc plays a vital role in insulin formation, stimulation of phosphorylation of the β-subunit of the insulin receptor, activation of PI3K, and GLUT4 translocation and expression in skeletal muscle (Zorzano et al., 2005; Kelishadi et al., 2010; Ranasinghe et al., 2015). Our data indicate that both Zn and implants influence serum insulin concentration in late-stage finishing steers. In rat L6 myotubes, Zn has been reported to influence glucose metabolism and uptake via Akt phosphorylation, GLUT4 translocation, and GSK3β phosphorylation (Wu et al., 2016). On day 20, increased insulin sensitivity, as indicated by RQUICKI index value, was accompanied by higher mRNA abundance for GLUT4 in TE200. This supports our hypothesis that increased anabolic stimulus from the steroidal implant would drive glucose uptake, and thus mRNA abundance for GLUT4.

The influence of supplemental ZnSO_4_ and IMP on key skeletal muscle Zn transporters was investigated. In this study, IMP did not influence any skeletal muscle Zn transporters of interest. On day 20, increasing the concentration of supplemental Zn resulted in increased skeletal muscle mRNA abundance of Zn transporter ZnT7. A similar numeric trend approaching significance was noted for ZnT5 on day 20 as well. Both ZnT5 and ZnT7 mediate the incorporation of Zn into enzymes, such as alkaline phosphatase, by the influx of Zn2+ into the lumen of the Golgi apparatus (Suzuki et al., 2005). A role for ZnT7 in other species has been reported where Huang et al. (2012) found signs of insulin resistance in skeletal muscle of ZnT7-KO mice.

Steroidal implants have been consistently shown to increase protein synthesis and influence the myogenic progression of satellite cells (Johnson et al., 1998; Reichhardt et al., 2021a). Satellite cells provide the additional nuclei necessary to support postnatal skeletal muscle fiber hypertrophy and are critical in determining the extent of muscle growth (Dayton and White, 2013). Differentiation of satellite cells is necessary for proper fusion to growing muscle (Halevy et al., 2004). Markers of differentiation in skeletal muscle include an increased abundance of MYF5, MYOD, and MYOG, as well as decreased paired box transcription factor 7 (**PAX7**) expression (Yin et al., 2013). The effects of Zn on myogenic markers of differentiation in both implanted and non-implanted cattle are poorly understood.

Day 20 represents a timepoint when the degree of implant payout and anabolic stimulus were near their peaks. In this study, on day 20, increased ZnSO_4_ supplementation increased mRNA abundance of AKT1 and mTOR, which are key regulators of protein synthesis and energy metabolism. Intracellular Zn influences the activation of PI3K/Akt signaling pathways (Nimmanon et al., 2017). Phosphorylated AKT elicits responses of several downstream signaling molecules, including mTOR, which upregulates protein synthesis.

Myogenic regulatory factor 5 is a myogenic regulatory factor. In bovine satellite cells induced to differentiate, MYF5 mRNA abundance is increased by TBA (Reichhardt et al., 2021b). On day 20 MYF5 mRNA abundance increased with increasing ZnSO_4_ supplementation and was greater in implanted cattle. No other myogenic regulatory factors were affected by ZINC or IMP on day 20.

Extracellular matrix factors such as matrix metalloproteinases (MMP) are essential for proper skeletal muscle growth. Matrix metalloproteinases are a family of Zn-containing enzymes implicated in the degradation and remodeling of the extracellular matrix (Ren et al., 2019). Expression of MMP2 on day 20 increased linearly with ZnSO_4_ supplementation. While the influence of Zn on MMP2 has not been previously assessed in beef cattle, prior work indicates that steroidal implants influence the mRNA abundance of both MMP2 and MMP9 (Reichhardt et al., 2021a). However, in this study, IMP did not affect MMP2 at either day 20 or 84. These day 20 gene expression results suggest Zn may influence implant-induced growth through factors related to protein synthesis, satellite cell progression, and extracellular matrix remodeling.

By day 84, no implant effects were noted on the expression of any genes, which fits with the lack of performance differences by this time. However, MYOD1 and MYOG mRNA abundance decreased with increasing ZnSO_4_. Myogenin differentiation factor 1 is a key regulator of myogenic cell differentiation. Roles for Zn in myogenic satellite cell progression have been documented (Paskavitz et al., 2018). Given the previously observed synergistic effects of Zn on implant-induced muscle growth, this result warrants further investigation. Analysis of skeletal muscle gene expression from both higher and lower growth potential cattle may help further refine and interpret the results reported herein.

## Conclusions

In this study, plasma and liver TM results were generally consistent with prior observations. However, Zn supplementation did not improve overall steroidal implant-induced growth performance or carcass characteristics. Unlike prior work, in this experiment, cattle were supplemented Zn for 60 d before implant and fed a basal diet containing nearly twice the current NASEM recommendations, resulting in highly adequate plasma Zn on day 0. Thus, basal diet Zn concentration and overall growth potential of cattle may be primary drivers that dictate the ultimate response to increased Zn supplementation in implanted cattle.

Our results support the theory that implant administration increases tissue demand for Zn during periods of rapid skeletal muscle growth. Based on RQUICKI index values, our data also indicate steroidal implants delay the decrease in insulin sensitivity which occurs later in the finishing period. This study also provides preliminary data outlining the influence of Zn supplementation and steroidal implants on mRNA abundance of skeletal muscle gene expression.

## Supplementary Material

skae154_suppl_Supplementary_Table

## Abbreviations

ADG: average daily gain
BW: bodyweight
DMI: dry matter intake
E_2_: estradiol
EBF: empty body fat
HCW: hot carcass weight
ICP-OES: inductively coupled plasma optical emission spectroscopy
KPH: kidney, pelvic, and heart fat
Mn: manganese
mRNA: messenger ribonucleic acid
NEFA: nonesterified fatty acid
SUN: serum urea nitrogen
TBA: trenbolone acetate
TM: trace mineral
ZIP: Zrt/Irt-like proteins (SLC39 family)
Zn: zinc
ZnT: Zn transporter (SLC30 family)
