# Supplementary material for: Influence of steroidal implants and zinc sulfate supplementation on growth performance, trace mineral status, circulating metabolites, and transcriptional changes in skeletal muscle of feedlot steers
Source: J Anim Sci. 2024 Jun 3;102:skae154. doi: 10.1093/jas/skae154 (PMC11190378; doi:10.1093/jas/skae154)
Supplement: skae154_suppl_Supplementary_Table [file skae154_suppl_supplementary_table.docx]

| **Supplemental Table 1.** Target gene primer information | | | |
| --- | --- | --- | --- |
| Target | Forward (5'-3') | Reverse (5'-3') | Design RefSeq |
| AKT1 | CGTGTCGGCCCTGGATTA | TGTCCAGCATGAGGTTCTCC | NM_173986.2 |
| AZGP1 | ACCAGGGGAAGTCCAGAACC | GACAGCCCGGTGTAGAGGAA | NM_001034331.1 |
| CAPN6 | AATCGTTCAGGAGGCTGCTA | TCATCCTCTGGCACAGTGAA | NM_001192231.1 |
| CKM | CTTCAAACCCACAGACAAGCA | CGTAGTTGGGGTCCAGATCA | NM_174773.4 |
| CKMT2 | GAGCGATTCTGTCGTGGACTTA | AGGCGCTCATTCCACATGAA | NM_001034656.2 |
| CS | CAGAGGGTATCAACCGAACCA | CACAAGGAAGCTTTGCGATCA | NM_001044721.1 |
| DCN | CCAAAGTTGATGCAGCTAGCC | ACCGCAGAGATGCTGTTGAA | NM_173906.4 |
| EEF1A2 | CGACAAGAGGACCATCGAGAA | TTGTCCAGTACCCAGGCATAC | NM_001037464.2 |
| MHCI | CTCTTCTGCGTCACCATCAAC | CCTCACTCCTCTTCTTGCCC | FLDM-401977.1 |
| IR | TGCACAACGTGGTTTTCATC | GTTTCCTCGAAGGCCTAGC | FLDM-455537.1 |
| MHCIIx | GCTCCTTACCTCCGAAAGTC | CTCTGCACAGTTGCTTTCAC | FLDM-816314.1 |
| mTOR | GTCAGCCTGTCAGAGTCCAA | GCAGGTTCCCCATGTTGAC | FLDM-995545.1 |
| FNDC5 | CTTGGAGGACGAGGTTGTCA | GGTGTTCACCTCCTGGATGAA | NM_001105421.1 |
| FOXO4 | GGTGCCCTACTTCAAGGACAA | ATAGGTTGTGGCGGATCGAA | NM_001101277.1 |
| GLUD1 | CCACAGCAGAGTTCCAAGACA | GCGCTCCATGGTGTAAGCTA | NM_182652.2 |
| GPX1 | CGGGACTACACCCAGATGAA | CTTCAGGCAATTCAGGATCTCC | NM_174076.3 |
| IGF1R | ACGGATCCCGTGTTCTTCTAC | AATGGGCAGAGCGATCATCA | NM_001244612.1 |
| LDHB | GAGGATTCACCCAGTGTCAAC | ATACACGGAAGGCTCAGGAA | NM_174100.2 |
| MEF2C | TCAGTAACTGGCTGGCAACA | TATCTCGAAGGGGTGGTGGTA | NM_001046113.1 |
| MMP2 | CCATGATGGAGAGGCTGACA | GCCCGTCTTTGCCATCAAA | NM_174745.2 |
| MMP9 | AGCACGCACGACATCTTTCA | GAACTCACGCGCCAGTAGAA | NM_174744.2 |
| MT2A | CGGCTCCTGCAAATGCAAA | CGAAGCCCCTTTGCAGAC | NM_001075140.1 |
| MYF5 | CACCAGCCCCACCTCAA | CAGGACAGTAGACGCTGTCA | NM_174116.1 |
| MYF6 | TCAGCTACAGACCCAAGCAA | CCCTGGAATGATCCGAAACAC | NM_181811.1 |
| MYH2 | ACCGTGAGGAATGACAACTCC | GCAGAGGCCAGTTTTCCTGTA | NM_001166227.1 |
| MYOD1 | ACTGCTACGACCGCACTTAC | ACCGCAGCGCTCTTCC | NM_001040478.2 |
| MYOG | CAGCGCACTGGAGTTTGG | AGGTGAGGGAGTGCAGATTG | NM_001111325.1 |
| NFE2L2 | TGAGCAAGTTTGGGAGGAAC | TAGTCTCAGCCAGCTTGTCA | NM_001011678.2 |
| NFKB2 | CTGACTGCACAGGATGAGAAC | ACGTGGGCTATCTGCTCAA | NM_001102101.1 |
| PARK7 | TGGCTAAAGGAGCAGAGGAAA | ACCTGCAACGGTGACCTTA | NM_001015572.1 |
| PAX7 | GCATCTTCCGAGACCAGTCC | CCCCAGGGACTTGAAGATGAA | FLDM-551832.1 |
| PDE4B | AGCCCCATGTGTGATAAGCA | CCCACAAGGGATGGACAATGTA | NM_001102546.1 |
| PDP1 | TGAGGACCAGAATGCAGCAA | AGAGGCGCTCATGATCAACA | NM_001206353.1 |
| PRKAA2 | AGCTGCGGATCCCCAAATTA | GCAGCTCCAGATATCCACTTCA | NM_001205605.1 |
| PYGM | CGCTGGTTGGTGATGTGTAA | TGATCCAGGTCTGCGATGTA | NM_175786.2 |
| RHEB | CCATCTTTCCTCAGACATACTCCA | CCCCACCATATCCAACAATTTACC | NM_001031764.2 |
| RPS9 | GCCTCGACCAAGAGCTGAA | GAATTTGACCCTCCAGACCTCA | NM_001101152.2 |
| RYR1 | TGAGGAGGAGAAGACGGAGAA | TTGTAGCCTTCCCTTGCATCA | NM_001206777.1 |
| SDHA | ACGCTCTCCTACGTTGACA | CGCAGTCAGTCTCGTTCAAA | NM_174178.2 |
| SLC2A4 | ATCATCGGCATGGGTTTCCA | GGAGGACCGCGAATAGAAGAA | NM_174604.1 |
| SLC30A5 | AGAGGCATGAACGCCAACA | GGTGGACACGATCACACCAA | NM_001192174.2 |
| **Supplemental Table 1.** Continued | | | |
| Target | Forward (5'-3') | Reverse (5'-3') | Design RefSeq |
| SLC30A7 | CTGGATTGGCAGCGTCTGTTA | AGCCAGAACCTCCGCTCTAA | NM_001083760.1 |
| SLC39A14 | TTTGCACTGGCTGGAGGAA | CTCTTGGCTCACCTCGTTCA | NM_001098036.1 |
| SLC39A7 | CATGCTCATGGTCACACACA | TCCTCTGAGCTCTGTTTCTCC | NM_001076237.3 |
| SLC39A8 | CCATCCTATGTGAGGAGTTCCC | GCTTGTCGAGTGCTCATTCC | NM_001205630.1 |
| SPARC | GACAGAAGTGGTGGAAGAAACC | TCCTCAGCACCATCATCGAA | NM_174464.2 |
| TRIM63 | AGTGCCAAGCAGCTCATCA | TCAAAGCCCTGCTCTATCTTCC | NM_001046295.1 |
